# Inhibition of Human Cytochrome P450 Enzymes by *Bacopa monnieri* Standardized Extract and Constituents

**DOI:** 10.3390/molecules19022588

**Published:** 2014-02-24

**Authors:** Seetha Ramasamy, Lik Voon Kiew, Lip Yong Chung

**Affiliations:** 1 Department of Pharmacy, Faculty of Medicine, University of Malaya, Kuala Lumpur 50603, Malaysia; E-Mail: seetah@hotmail.com; 2 Department of Pharmacology, Faculty of Medicine, University of Malaya, Kuala Lumpur 50603, Malaysia; E-Mail: lvkiew@um.edu.my

**Keywords:** *Bacopa monnieri*, bacoside, cytochrome P450 (CYP), herb-drug interactions, Scrophulariaceae

## Abstract

*Bacopa monnieri* and the constituents of this plant, especially bacosides, possess various neuropharmacological properties. Like drugs, some herbal extracts and the constituents of their extracts alter cytochrome P450 (CYP) enzymes, causing potential herb-drug interactions. The effects of *Bacopa monnieri* standardized extract and the bacosides from the extract on five major CYP isoforms *in vitro* were analyzed using a luminescent CYP recombinant human enzyme assay. *B. monnieri* extract exhibited non-competitive inhibition of CYP2C19 (IC_50_/Ki = 23.67/9.5 µg/mL), CYP2C9 (36.49/12.5 µg/mL), CYP1A2 (52.20/25.1 µg/mL); competitive inhibition of CYP3A4 (83.95/14.5 µg/mL) and weak inhibition of CYP2D6 (IC_50_ = 2061.50 µg/mL). However, the bacosides showed negligible inhibition of the same isoforms. *B. monnieri*, which is orally administered, has a higher concentration in the gut than the liver; therefore, this herb could exhibit stronger inhibition of intestinal CYPs than hepatic CYPs. At an estimated gut concentration of 600 µg/mL (based on a daily dosage of 300 mg/day), *B. monnieri* reduced the catalytic activities of CYP3A4, CYP2C9 and CYP2C19 to less than 10% compared to the total activity (without inhibitor = 100%). These findings suggest that *B. monnieri* extract could contribute to herb-drug interactions when orally co-administered with drugs metabolized by CYP1A2, CYP3A4, CYP2C9 and CYP2C19.

## 1. Introduction

*Bacopa monnieri* (Linn.) Pennell (Scrophulariaceae), also known as brahmi in Ayurvedic medicine, has been used in traditional and Ayurvedic medicine for centuries as a brain tonic to enhance learning and memory and to improve concentration [1]. These traditional claims have been supported by several preclinical and clinical studies [2,3,4,5], and the observed cognitive effects have been attributed to bacoside A [6,7,8], which is a mixture of four triglycosidic saponins (bacoside A3, bacopaside II, bacopaside X and bacopasaponin C) [9]. A reputable nootropic agent and the second most highly touted herb in Ayurveda [10,11], *B. monnieri* is widely marketed and used not only in India but also in the international market. Since *B. monnieri* is used as a neuropharmacological agent [1,10], the chances of chronic or recurrent usage of *B. monnieri*-related products by patients with mental illnesses are very high. These patients are also most likely to be prescribed therapeutic drugs, raising the potential for herb-drug interactions. 

Drug interactions can lead to serious adverse events or decreased drug efficacy. These interactions may occur through the inhibition or induction of hepatic and intestinal drug-metabolizing enzymes (e.g., CYPs) and transporters (e.g., p-glycoprotein) [12]. CYP-mediated drug interactions are a major concern because CYP enzymes are involved in phase 1 metabolism of more than 70% of prescription drugs [13]. Inhibition of a CYP enzyme causes an increase in drug plasma levels through decreased drug metabolism, which could result in significant adverse reactions or toxicity. The induction of CYP enzymes leads to a decreased drug plasma and drug efficacy. Therefore, inhibition-based drug interactions are a primary cause of clinically significant drug interactions [14].

The major CYPs involved in the hepatic metabolism of most drugs include CYP1A2, CYP3A4, CYP2C9, CYP2C19, CYP2D6 and CYP2E1 [14]. CYPs are also expressed in extrahepatic tissues, such as the intestinal mucosa, kidneys, lungs, skin and brain [15]. Among these tissues, the enzymes in the intestinal mucosa are the most important contributors in drug metabolism [16]. The CYPs in the small intestine were characterized by Paine *et al.* [17], and CYP3A4 was found to be the most abundant CYP enzyme (~80%), followed by CYP2C9 (~15%), CYP2C19 (2%), CYP2J2 (<2%) and CYP2D6 (<1%). Since herbs are often orally administered, the high concentration of herbal constituents in the gut lumen may potentially affect the intestinal enzymes activity. This effect could enhance or reduce the bioavailability of co-administered drugs, resulting in clinically important herb-drug interactions. For example, grapefruit juice is known to inhibit intestinal CYP3A4 and causes an increase in the bioavailability of drugs that are substrates of CYP3A4 [18]. 

**Figure 1:**
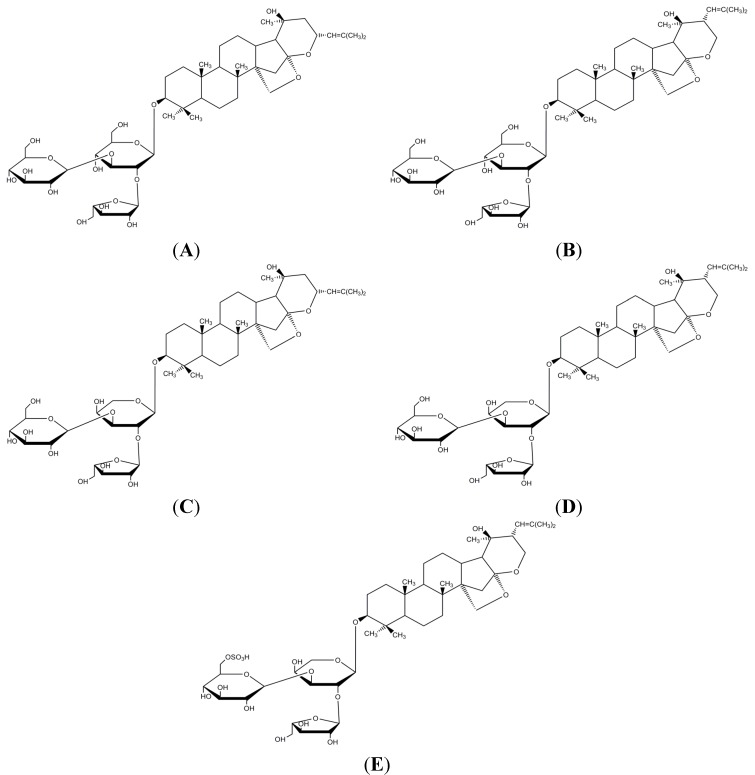
Structures of *B. monnieri* constituents, (**A**) bacoside A3; (**B**) bacopaside II; (**C**) bacopaside X; (**D**) bacopasaponin C and (**E**) bacopaside I. Bacoside A is a mixture of components A, B, C and D. These bacosides are dammarane-type triterpenoid saponins that have three sugar chains linked to a nonpolar triterpene aglycone skeleton.

*B. monnieri* products are classified as herbal or natural products, and in most countries, the regulatory requirements to market natural products are less stringent compared to conventional drugs because natural products are considered to be low risk products [19]. However, natural products contain a complex mixture of active phytochemicals that could alter enzymatic systems, transporters and other physiologic process [20]. Therefore, like drugs, herbal extracts such as *B. monnieri* that show promising results in clinical trials [4,5] should also be tested for herb-drug interactions before the extracts are marketed for therapeutic use. Furthermore, the widespread use of *B. monnieri* products and the lack of information on the effect of *B. monnieri* extract and extract constituents on CYP enzymes warrant the study of this extract and its constituents on human CYP enzymes. In this study, *B. monnieri* standardized methanol extract and some of the reportedly active and commercially available constituents, including bacoside A, bacoside A3, bacopaside II, bacopaside X, bacopasaponin C and bacopaside I (Figure 1), were chosen to determine the inhibitory effects on five major CYP isoforms, CYP1A2, CYP3A4, CYP2C9, CYP2C19 and CYP2D6. 

## 2. Results

The inhibitory effects of *B. monnieri* standardized extract and the constituents bacoside A, bacoside A3, bacopaside II, bacopaside X, bacopasaponin C and bacopaside I on human cytochrome P450 enzyme were examined using an *in vitro* luminescent assay. The P450-Glo™ substrates are converted by CYP enzymes to a luciferin product that reacts with a Luciferin Detection Reagent to produce light. The amount of light produced is directly proportional to the CYP enzyme activity. The net signals from untreated (added with buffer or solvent) CYP reactions represent total CYP activity (without any inhibition = 100%). The modulation of the CYP activity by the test compound was determined by comparing the changes from the average net signal of untreated CYP reactions with the changes observed due to the test compound. The changes were typically observed as decreases due to CYP inhibition. The test compounds that inhibit CYP enzymes caused a reduction in CYP activity and therefore generated less light/signal.

### 2.1. The Determination of the Apparent Half-Maximal Inhibitory Concentration (IC_50_) for Test Samples and Standard Inhibitors

**Figure 2:**
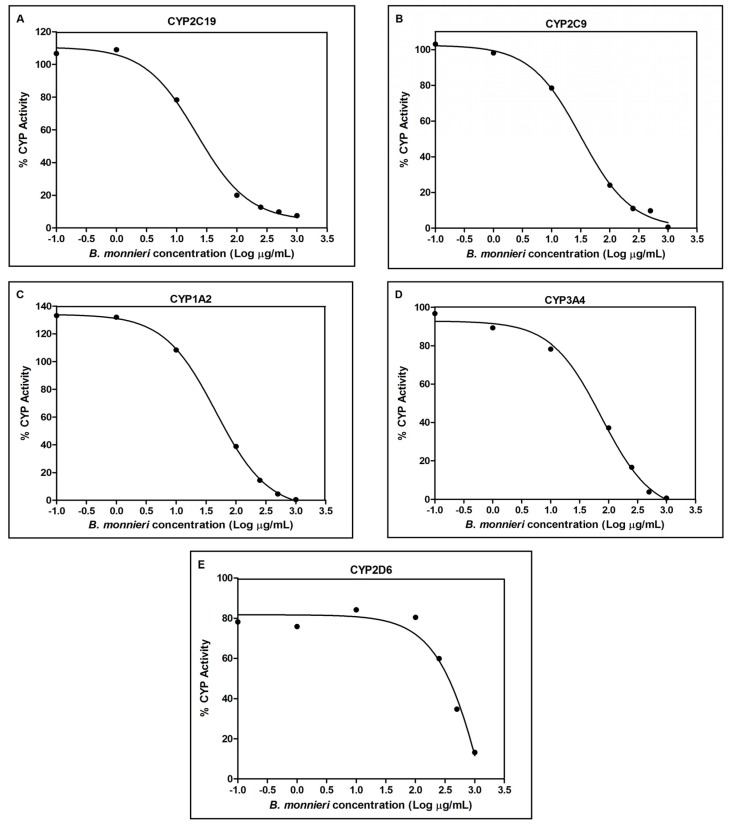
*B. monnieri* extract showed strongest inhibitory effects towards CYP2C19 (**A**, IC_50_ = 23.67 µg/mL) followed by CYP2C9 (**B**, 36.49 µg/mL), CYP1A2 (**C**, 52.20 µg/mL), CYP3A4 (**D**, 83.95 µg/mL) and CYP2D6 (**E**, IC_50_ = 2061.50 µg/mL). Each point represents the average of triplicate determinations.

The inhibitory potencies of *B. monnieri* extract and the constituents against CYP450 were determined by evaluating the IC_50_ values. According to Kong *et al.* [21], the potency of a test compound can be classified according to its IC_50_ values, as potent, if IC_50_ ≤ 20 μg/mL or ≤10 μM, moderate if IC_50_ 20–100 μg/mL or 10–50 μM, or weak if IC_50_ ≥ 100 μg/mL or ≥50 μM. All positive controls were found to show potent CYP450 inhibition and the IC_50_ values were consistent with previously reported values [22,23,24]. *B. monnieri* extract was found to exhibit moderate inhibition against CYP2C19, CYP2C9, CYP1A2, and CYP3A4 and weak inhibitory activities against CYP2D6 (Table 1 and Figure 2), with most potent inhibition on CYP2C19 (IC_50_ = 23.67 ± 2.84 µg/mL). However, all of the constituents, bacoside A, bacoside A3, bacopaside II, bacopaside X, bacopasaponin C and bacopaside I at concentrations up to 100 µM, showed negligible inhibition towards the five CYP enzymes (Table 1).

**Table 1:** *B. monnieri* extract, but not its identified constituents, inhibit CYP450 enzymes in a moderate fashion. Inhibition on CYP2C19 was found to be most potent.

Test Compounds	IC_50_ for CYP450 enzyme inhibition assays (µg/mL) ^a^				
	CYP1A2	CYP3A4	CYP2C9	CYP2C19	CYP2D6
*B. monnieri* ext.	52.20 ± 8.46	83.95 ± 12.97	36.49 ± 6.60	**23.67 ± 2.84**	2061.50 ± 173.24
bacoside A	>76.90	>76.90	>76.90	>76.90	>76.90
bacoside A3	>92.91	>92.91	>92.91	>92.91	>92.91
bacopaside II	>92.91	>92.91	>92.91	>92.91	>92.91
bacopaside X	>89.91	>89.91	>89.91	>89.91	>89.91
bacopasaponin C	>89.91	>89.91	>89.91	>89.91	>89.91
bacopaside I	>97.91	>97.91	>97.91	>97.91	>97.91
Positive control	0.78 ± 0.02	0.32 ± 0.21	0.03 ± 0.01	7.81 ± 0.26	0.02 ± 0.01
(compound)	(α-naphthoflavone)	(ketoconazole)	(sulfaphenazole)	(troglitazone)	(quinidine)

^a^ Values are expressed as the means ± S.D. of three determinations with two independent experiments.

Since the concentration of orally administered *B. monnieri* is likely to be higher in the gut than the liver, we sought to estimate the inhibitory effects of this extract on the CYPs present in the intestine, CYP3A4, CYP2C9 and CYP2C19, at a gut concentration estimated according to calculation method described by Fotti *et al.* [25]. Theoretically, a daily recommended *B. monnieri* extract dose of 300 mg/day [2,3,4,26] and an intestinal volume of approximately 500 mL may result in an estimated *B. monnieri* gut concentration of 600 μg/mL (Table 2). At this concentration, *B. monnieri* extract may potentially reduce the catalytic activities of CYP3A4, CYP2C9 and CYP2C19 to less than 10% of the total CYP activity (without any inhibition ~100%, from Figure 2). Therefore, *B. monnieri* extract at the estimated gut concentration could increase the bioavailability of any therapeutic drug orally co-administered with it, if intestinal CYP450 plays significant roles in the degradation of these drugs.

**Table 2:** Suppression of intestinal CYP450 activities by recommended oral dose of *B. monnieri* extract (300 mg/day). The projected % CYP450 activity values were calculated based on the theoretical model proposed by Fotti *et al.* (2007).

Daily recommended dose ^a^	Estimated gut concentration ^b^	CYP enzyme	%CYP activity after inhibition ^c^
		CYP3A4	4.1
300 mg/day	600 μg/mL	CYP2C9	4.9
		CYP2C19	7.9

^a^ Daily recommended dose for *B. monnieri* used in clinical trials [2,3,4,26]; ^b^ Estimated gut concentration range was calculated using the daily recommended dose divided by 500 mL [25]; ^c^ Determined by comparing the changes from the average net signal of untreated CYP reactions [represent total CYP activity = 100% (without inhibition)] with the changes observed due to *B. monnieri* extract (from Figure 2).

### 2.2. Determination of the Inhibition Constant (Ki) Values and the Modes of Inhibition of B. monnieri Extract

**Figure 3:**
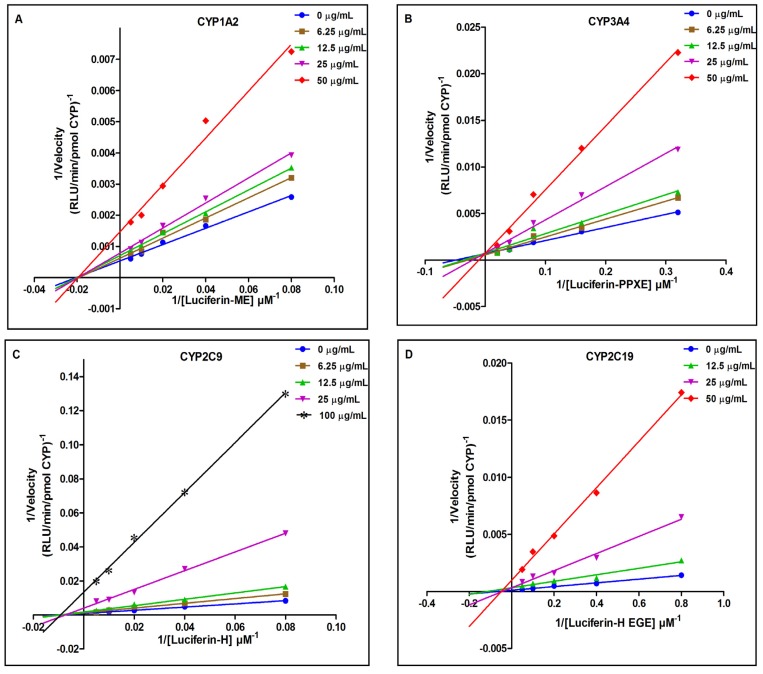
*B. monnieri* extract non-competitively inhibited CYP1A2, CYP2C9 and CYP2C19 activity and competitively inhibited CYP3A4 activity, as demonstrated by Lineweaver-Burk plots (**A**) CYP1A2; (**B**) CYP3A4; (**C**) CYP2C9 and (**D**) CYP2C19. The mode of inhibition was determined by incubating different concentrations of *B. monnieri* extract with increasing concentrations of substrates. Each point represents the average of duplicate measurements.

We further characterized the CYP450 inhibitory properties of *B. monnieri* extract by determining the Ki value (the binding affinity of the inhibitor for an enzyme) and investigating the possible mode of inhibition for *B. monnieri* extract on the activities of human CYP1A2, CYP3A4, CYP2C9 and CYP2C19 isoforms (Table 3 and Figure 3). 

**Table 3:** Inhibition constant (Ki) and modes of inhibition for *B. monnieri* extract on the activities of human CYP enzymes.

CYP enzyme	Ki (μg/mL) ^a,b^	Mode of inhibition
CYP1A2	25.1	Non-competitive
CYP3A4	14.5	Competitive
CYP2C9	12.5	Non-competitive
CYP2C19	9.5	Non-competitive

^a^ Each value represents the average of duplicate measurements; ^b^ Ki values are derived from the secondary plots of respective CYP activity using the slopes of the primary Lineweaver-Burk plots *versus* *B. monnieri* extract concentration.

**Figure 4:**
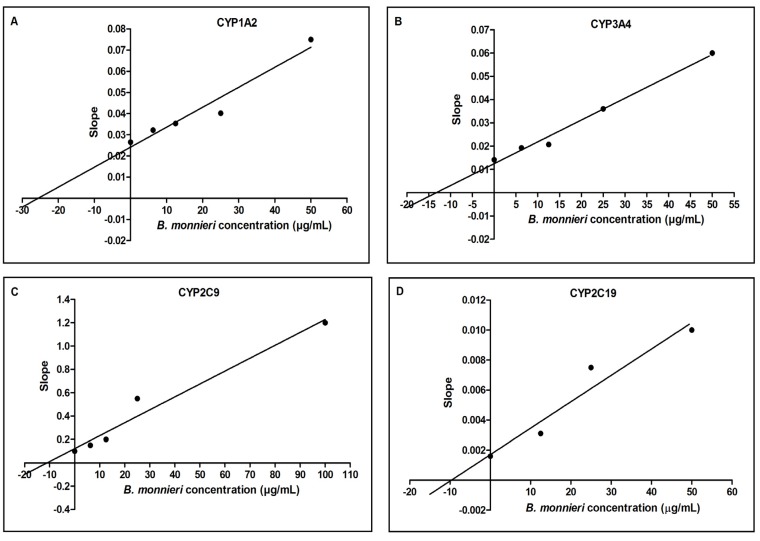
Ki values of *B. monnieri* extract for (**A**) CYP1A2; (**B**) CYP3A4; (**C**) CYP2C9 and (**D**) CYP2C19. Ki values were determined from secondary plots of CYP activity using the slopes of primary Lineweaver–Burk plot *versus* concentrations of *B. monnieri* extract. Kinetic assays were carried out in the same conditions described in Figure 3. *B. monnieri* Ki values for CYP1A2, CYP3A4, CYP2C9 and CYP2C19 are of 25.1, 14.5, 12.5, and 9.5 μg/mL, respectively. Each point represents the average of duplicate measurements.

The Lineweaver-Burk double reciprocal plot shows that *B. monnieri* extract non-competitively inhibited CYP1A2, CYP2C9 and CYP2C19 activity and competitively inhibited CYP3A4 activity (Figure 3). During non-competitive inhibition, the substrate and the inhibitor concurrently attach to the enzyme at different sites. During competitive inhibition, the substrate and the inhibitor compete to bind at the same active site of the enzyme. Therefore, increasing the substrate concentration does not decrease inhibition during non-competitive inhibition but can decrease inhibition during competitive inhibition. Hence, both type of inhibition will result in elevated plasma levels of therapeutic drugs that are substrates of these CYPs if taken with *B. monnieri*. However, if the drug concentration is higher, the competitive inhibition of CYP3A4 can be decreased, but not for CYP1A2, CYP2C9 and CYP2C19 inhibition. The secondary plots based on the slope of Lineweaver-Burk plots show that the Ki values for CYP1A2, CYP3A4, CYP2C9 and CYP2C19 are of 25.1, 14.5, 12.5, and 9.5 μg/mL, respectively (Figure 4). Similar to the results of the IC_50 _ analysis, *B. monnieri* was found to most potently inhibit CYP2C19, followed by CYP2C9.

## 3. Discussion

The modulation of CYP activity by *B. monnieri* extract and bacosides (bacoside A, bacoside A3, bacopaside II, bacopaside X, bacopasaponin C and bacopaside I) were studied using recombinant human CYP1A2, CYP3A4, CYP2C9, CYP2C19 and CYP2D6 enzymes. The pharmacokinetic parameters (IC_50_, Ki values) show that *B. monnieri* extract most strongly inhibits CYP2C19 followed by CYP2C9, CYP1A2, and CYP3A4 and most weakly inhibits CYP2D6. However, all of the bacosides showed negligible inhibition towards all five CYP enzymes. Bacosides are dammarane-type triterpenoid saponins that have one or more sugar chains linked to a nonpolar triterpene aglycone skeleton. Due to the presence of three glycosides in the bacoside structure, these molecules have low log *P* values and a high number of hydrogen bond acceptors and donors. The high polarity and the low log *P* value of bacosides could result in low affinities to CYP active sites and negligible inhibition of the CYP isoforms. These results are similar to the CYP inhibitory activity of other triterpenoid saponins such as asiaticoside [27] and ginsenosides [28]. Pan *et al.* [27] reported that asiaticoside had high IC_50_ values, indicating negligible or low potential for asiaticoside to modulate CYP2C9, CYP2D6 and CYP3A4 enzymatic activity. Of the seven ginsenosides tested on the catalytic activity of *c*DNA expressed CYPs (CYP1A2, CYP2C9, CYP2C19, CYP2D6 and CYP3A4), only one showed weak inhibition of CYP3A4 and CYP2D6. The author suggested that the tested ginsenosides are not likely to inhibit drug metabolizing enzymes and would not inhibit the metabolism of co-administered drugs that are primarily eliminated through CYP450. The above result implies that the tested bacosides are not responsible for the inhibition of the CYP isoforms by *B. monnieri* extract. The inhibition of the CYP enzymes by *B. monnieri* extract could be due to the presence of other constituents in the extract. This could include free aglycones, such as jujubogenin [29] and pseudojujubogenin [30], which are more lipophilic. Free aglycones that are more lipophilic and are better able to hydrogen bond bind more readily to the CYP isoforms, resulting in stronger inhibitory effects [27].

Although the IC_50_ values for *B. monnieri* extract suggest moderate inhibition of the CYPs present in the liver, a significant amount of CYPs, especially CYP3A4, are also present in the intestine. Since *B. monnieri* is administered orally, a high concentration of *B. monnieri* in the gut might cause significant inhibition of CYP3A4, CYP2C9 and CYP2C19 and reduce the activity of these enzymes to less than 10% of the total activity. Therefore, the concomitant use of *B. monnieri* with clinically prescribed drugs that are substrates of CYP3A4, CYP2C9, and CYP2C19 isoforms, particularly those exhibiting poor bioavailability due to extensive metabolism such as clozapine [31] and midazolam [32] could cause potential herb-drug interactions. Due to the traditional popularity of *B. monnieri* as a brain tonic and the suggested use for Alzheimer’s disease (AD) [33], anxiety [34], depression [35] and epilepsy [36], mental disorder patients who are on prescription drugs might use *B. monnieri* as an alternative medicine, and some prescription drugs that are used for AD, anxiety, depression and epilepsy are metabolized by CYP2C19, CYP2C9, CYP1A2, and CYP3A4. 

CYP2C19 is involved in the metabolism of centrally active antidepressants, including citalopram, clomipramine, imipramine; anxiolytics, such as diazepam and alprazolam; and anticonvulsants, such as phenobarbital [37]. Drugs that are substrates for CYP2C9 include antidepressants, such as amitriptyline and fluoxetine, and antiepileptics, such as phenytoin [38]. CYP1A2 is also involved in the metabolism of antidepressants drugs, such as amitriptyline, clomipramine, fluvoxamine, and imipramine, and antipsychotic drugs, such as clozapine, chlorpromazine and haloperidol [38]. CYP3A4 is the most abundant enzyme and is involved in the metabolism of nearly 50% of clinically available drugs, including psychotropics and anticonvulsants. Because *B. monnieri* extract inhibits CYP2C19, CYP2C9, CYP1A2, CYP3A4, patients taking *B. monnieri* with the drugs mentioned above could experience an increase in the plasma level of the drugs, which could result in significant adverse reactions or toxicities. 

Furthermore, CYP2C19 and CYP2C9 exhibit genetic polymorphisms [39]. In a general population, an “extensive metabolizer” (i.e., normal) has two copies of wild-type alleles. “Poor metabolizers” have two copies of variant alleles, causing reduced enzymatic activity, whereas “ultrarapid metabolizers” inherit multiple copies of wild-type alleles which results in excessive enzyme activity [40]. Ultra extensive metabolism can cause therapeutic failure due to reduced bioavailability or lack of drug activation, and poor metabolism can lead to drug toxicity and possibly death. Thus, further inhibition of CYP2C19 and CYP2C9 by *B. monnieri* could result in clinically important herb-drug interactions in individuals who are already “poor metabolizers”. In the light of this, further investigation on the *in vivo* drug-herb interaction between *B. monnieri* extracts with the abovementioned drugs may be of importance.

## 4. Experimental

### 4.1. Chemicals and Reagents

The assay was carried out using P450-Glo™ Screening System (Cat.# V9770, V9790, V9880, V9890, V9910) from Promega (Madison, WI, USA). The system includes a membrane preparation containing recombinant human cytochrome P450 (CYP) enzyme, negative control membranes, a luminogenic substrate appropriate for the CYP enzyme, an NADPH regeneration system (containing nicotinamide adenine dinucleotide phosphate (NADP^+^), glucose-6-phosphate, magnesium chloride (MgCl_2_) and glucose-6-phosphate dehydrogenase functioning to initiate and sustain the CYP450 reaction by maintaining a non-limiting NADPH system), reaction buffer, Luciferin Detection Reagent and Luciferin-Free Water. The membranes were prepared from baculovirus-infected insect cells and contained recombinant human CYP enzyme and P450 reductase (and cytochrome b5 for CYP2C9, 2C19 and 3A4). The negative control membranes were devoid of CYP activity. The dried powder of *B. monnieri* standardized methanolic extract containing 50% bacosides (batch No.: C92352/H) was purchased from Sami Labs Ltd. (Karnataka, India). Bacoside A, bacoside A3, bacopaside II, bacopaside X, bacopasaponin C and bacopaside I were purchased from Chromadex Inc. (Irvine, CA, USA). The positive controls quinidine, ketoconazole, α-naphthoflavone, troglitazone and sulfaphenazole were purchased from Sigma Aldrich (St. Louis, MO, USA).

### 4.2. Enzyme Assay

The enzyme assay was performed in 96-well white bottom flat plates (Nunc, Roskilde, Denmark). The concentration of *B. monnieri* extract was 0.01–1000 µg/mL, and the positive controls and compounds (bacoside A, bacoside A3, bacopaside II, bacopaside X, bacopasaponin C and bacopaside I) were 0.01–100 µM. The concentration of the extracts, compounds and positive controls were prepared at 4× the original concentration. All extracts and compounds were dissolved in dimethyl sulfoxide (DMSO). The organic solvent was kept below 0.25% (v/v) in the incubation mixture. First, 12.5 μL of the 4× test compounds or positive controls (appropriate for each enzyme) were added to the “treated” wells. For the “untreated” (the values from these wells represent total CYP activity) and “minus-P450 control” wells (the values from these wells represent the CYP-independent background luminescence of the assay), 12.5 μL of vehicle (1% DMSO) was added. Then, 12.5 μL of the 4× control reaction mixture (containing membrane preparations devoid of CYP enzymes, the appropriate luminogenic substrate, and potassium phosphate buffer) were added to the minus-P450 control wells, and 12.5 μL of the 4× reaction mixture (containing human CYP membrane preparations, the appropriate luminogenic substrate, and potassium phosphate buffer) were added to all other wells. The plate was pre-incubated at room temperature for 10 min, and then 2× NADPH regeneration system was added to initiate the reaction. After incubation at room temperature for 30 min (45 min for CYP2D6), 50 μL of the reconstituted Luciferin Detection Reagent was added to stop the reaction and generate the luminescent signal. Before reading, the plate was incubated at room temperature for 20 min to stabilize the luminescent signal. The luminescence in all of the samples was measured using an Infinite F200 plate reader (Tecan, Männedorf, Switzerland). The values were displayed as relative light units (RLU). A summary of the reaction components and the assay conditions are listed in Table 4.

The percentage of CYP enzyme activity *versus* log concentration of test compounds were plotted to calculate the IC_50_ values. Ki values and mode of inhibition were further determined for those with IC_50_ less than 100 μg/mL (for extracts) or 100 μM (for active constituents). Ki and mode of inhibition were determined by incubating a series of *B. monnieri* extract concentration with different concentrations of respective substrates for each CYP. 

**Table 4:** Summary of reaction components and assay conditions for the luminescence CYP inhibition assays.

	CYP1A2	CYP3A4	CYP2C9	CYP2C19	CYP2D6
Substrate ^a,^* (Concentration) ^b^	Luciferin-ME (100 μM)	Luciferin-PPXE (25 μM)	Luciferin-H (100 μM)	Luciferin-H EGE (10 μM)	Luciferin-ME EGE (30 μM)
Enzyme ^b^	0.5 pmol	0.5 pmol	0.5 pmol	0.25 pmol	0.25 pmol
Potassium Phosphate ^b^	100 mM	200 mM	25 mM	50 mM	100 mM
Incubation Time	30 min	30 min	30 min	30 min	45 min
Temperature ^c^	RT	RT	RT	RT	RT

^a^ Luciferin-ME = luciferin 6-methyl ether; Luciferin-PPXE = luciferin 6-phenylpiperazinylyl; Luciferin-H = 6-deoxyluciferin; Luciferin-H EGE = ethylene glycol ester of 6-deoxyluciferin; Luciferin-ME EGE = ethylene glycol ester of luciferin 6-methyl ether; ^b^ Concentration listed are final concentration of components per well; ^c^ RT = room temperature between 20–25 °C; * Substrate concentration = apparent K_m_ values. Percentage of substrate consumed by the end of incubation in control assay (without inhibitor) is approximately 60% (CYP1A2); 75% (CYP3A4); 40% (CYP2C9); 55% (CYP2C19) and 45% (CYP2D6).

### 4.3. Data Analysis

Prism Version 5.02 (GraphPad Software Inc., San Diego, CA, USA) software was used to calculate the IC_50_ values by non-linear regression analysis. The mode of inhibition was determined graphically from the Lineweaver-Burk plots. The Ki values were determined using the secondary plots constructed based on the slope of Lineweaver-Burk plots.

## 5. Conclusions

In summary, *B. monnieri* standardized extract inhibited CYP enzymes, but the constituents bacoside A, bacoside A3, bacopaside II, bacopaside X, bacopasaponin C and bacopaside I showed negligible inhibition. From the IC_50_ and Ki values, the results indicate that *B. monnieri* extract moderately inhibited CYP2C19, CYP2C9, CYP1A2, and CYP3A4, but weakly inhibited CYP2D6. Competitive inhibition was observed for CYP3A4, and non-competitive inhibition was observed for CYP2C19, CYP2C9 and CYP1A2. Furthermore, at estimated gut concentrations, *B. monnieri* showed potent inhibitory effects towards the intestinal CYPs. Hence, the pre-systemic herb-drug interaction through intestinal CYP3A4, CYP2C9 and CYP2C19 should be considered a possibility. The compounds that are responsible for the inhibition of the CYPs remain unknown and require further work. Co-administration of *B. monnieri* preparations with drugs that are primarily cleared via CYP2C19, CYP2C9, CYP1A2, CYP3A4 catalyzed metabolism should be performed with caution.
